# Moral and social reasons to acknowledge the use of cognitive enhancers in competitive-selective contexts

**DOI:** 10.1186/s12910-016-0102-8

**Published:** 2016-03-29

**Authors:** Mirko D. Garasic, Andrea Lavazza

**Affiliations:** Edmond J. Safra Center for Ethics, Tel Aviv University, Tel Aviv, Israel; Centro Universitario Internazionale, Arezzo, Italy

**Keywords:** Cognitive enhancement, Privacy, Performance-enhancing substances, Moral duty, Autonomy, Public interest

## Abstract

**Background:**

Although some of the most radical hypothesis related to the practical implementations of human enhancement have yet to become even close to reality, the use of cognitive enhancers is a very tangible phenomenon occurring with increasing popularity in university campuses as well as in other contexts. It is now well documented that the use of cognitive enhancers is not only increasingly common in Western countries, but also gradually accepted as a normal procedure by the media as well. In fact, its implementation is not unusual in various professional contexts and it has its peak in colleges (where the trend has been characterized as “academic doping”). Even when certain restrictions in the legislation of a country are indeed in place (i.e. through prescriptions requirements), they are without doubts easy to overcome. The legitimacy and appropriateness of such restrictions will not be the focus of our investigation.

**Discussion:**

Our concern is instead related to the moral and social reasons to publicly acknowledge the use of cognitive enhancers in competitive-selective contexts. These reasons are linked to a more neutral analysis of contemporary Western society: it is a fact that an increasing number of competitive-selective contexts have a substantial number of contenders using cognitive enhancers.

**Summary:**

Through the use of five explicative examples, in this paper we want to analyse the problems related to its use. In particular, it will be our aim to show the tension between one of the main argument used by bio-liberals (the use of cognitive enhancers is an eligible procedure that society does not impose on anyone) and the actual implementation of the drugs in competitive, or semi-competitive contexts.

## Background

The use of cognitive enhancers seems to be growing in several competitive contexts, such as schools, universities, firms and even the army (though its use in the latter context is certainly less surprising as soldiers have been historically prone to function as “guinea pigs” for a number of enhancers destined to eventually benefit civilians [1, 2]. This phenomenon has triggered a significant debate in philosophy and bioethics [3–8], and has received an increasing attention from the media, a fact that highlights the social relevance of the issue [9–14]. The situation is made even more complex by the non-univocal definition of what an enhancer is and, more specifically, what a *cognitive enhancer* is. In general, Cognitive Enhancers (CE) are not new to our society. Caffeine and nicotine are largely accepted as legitimate ways to help us be more focused, stay awake and be more productive. But meditation, yoga and a good night sleep are also things we could do to enhance our performance. So how to define a cognitive enhancer?

It seems reasonable to stress that there are substantial differences between the *natural* enhancers we just mentioned and the newer ones, which this paper intends to focus on. First of all, large doses of caffeine and nicotine do not provide the high level of focus that would be typical of specific modern CE – in other words, the “peak” of the performance is not comparable to that sought through the use of methylphenidate and Modafinil, for instance [15–17]. It should be pointed out that, despite their increasing use, there is still not robust evidence that methylphenidate and Modafinil provide effective cognitive enhancement [18–21]. Yet, given the demand and the ongoing research, it is not unreasonable to assume that similar effective drugs will be available soon.

Furthermore, the common and prescription-free availability of caffeine and nicotine might suggest that an implicit moral assessment over their use has already passed the scrutiny of Western society, at least in terms of their enhancing features, while the same cannot be said of the new ones. However, this is not the generally used criterion. For instance, Khat (a natural stimulant drug) does not require prescription, although its different status is due to the fact that no moral assessment has been conducted in Western societies – perhaps also because of the “cultural aspect” related to it, since certain ethnic groups traditionally use it [22]. In contrast, intensive use of caffeine and, obviously, nicotine has been discouraged mainly for medical reasons or, more rarely, in order to contrast a more general inclination towards vices [23–25]. Yet, the use of caffeine and nicotine is not reproached in competitive contexts.

Here our aim is to focus on a specific category of enhancers, which, as we shall see, might lead to several problems if their use were widespread. For those enhancers, therefore, there might be the need to set rules and boundaries, both moral and legal. We are referring to Pharmaceutical Cognitive Enhancers (PCE): substances able to improve some cognitive functions due to their action on the biochemical balance of the brain [26–28]. More specifically, PCE may act on the subject’s attention, alertness, executive functioning (planning, problem-solving and inhibition), memory and learning. In this way, PCE can improve performance in study and work by increasing concentration, motivation and accuracy. The substances most commonly included in the set of PCE are drugs used off-label by healthy people who do not have specific deficits but want to improve their standards of intellectual and cognitive performance [29–36]. Recent studies have focused particularly on Modafinil [21, 37], stressing how its enhancing nature at neurological level differs significantly from that of substances such as nicotine or caffeine. The substances that are currently most widely used (and deemed to be the most effective) are those marketed for the treatment of neurodegenerative disorders, ADHD and narcolepsy [38–40]. Thus, caffeine and nicotine, as well as other forms of enhancement, are excluded from the domain of PCE. The argument we will propose, however, is likely to be extended to several forms of cognitive enhancement, whether chemical or in the form of technological devices or implants.

PCE can be acquired through unofficial or illegal channels, overcoming the restrictions that many countries have set on their sale. For example, a report published in August 2015 [41] by the Federal Substance Abuse and Mental Health Services Administration affirms that roughly 137,000 American college students start *abusing* prescription stimulants each year. Moreover, the report shows a peak usage of such substances (no specific PCE are named) in periods of the year associated with key moments in the academic year. The legitimacy and appropriateness of such restrictions has been discussed elsewhere [42, 43] and will not be the focus of the present investigation. Our concern here is different. Our premise is that PCE use, in principle, could be regarded as legally permissible and morally acceptable.

Currently, the United Nations Convention on Psychotropic Substances [44] “defines the schedules for potentially dangerous psychotropic substances and explicitly lists methylphenidate as a Schedule II drug (which includes controlled substances with known medical uses). All countries that have ratified this Convention are obligated to regulate methylphenidate accordingly. Thus, the legal sanctions related to ‘study aid’ use, depending on the jurisdiction, might even include liability to imprisonment if methylphenidate is sold to, or shared with, others. Modafinil did not exist when the international legal framework was established and is not mentioned in the Convention’s schedules. Partly as a consequence of this, the controls around Modafinil vary enormously from country to country” [45].

Thus, the legal permissibility of these drugs could be supported by a liberal principle whereby the State should not interfere with its adult citizens’ choices as long as they do not damage third parties. In addition to this, it can be argued that cognitive performance enhancement is usually both an individual choice and a contribution to the wellbeing of society. The latter is obtained, for instance, through the enhancement of some professional performances, which could be an advantage for everyone. For example, surgeons could improve their practices and outcomes thanks to enhanced attention, working memory and self-control [46].

Moral acceptability, on the other hand, is connected to at least two general arguments. First of all, PCE are not qualitatively different from other common elements (from social contexts to schools of excellence) that enhance cognitive performance. These methods – even though potentially available to everyone – are as unequally distributed and accessible as PCE. Our second argument for moral acceptability has to do with the liberal perspective (prevailing today for a range of reasons that we cannot analyse here) which favours individual autonomy and self-determination. Within such a political scheme (the assessment of the validity of such a political approach goes beyond the scope of this paper) that allows -and to an extent encourages- various methods of self-creation, we argue that PCE do not constitute a less morally acceptable method than other individuo-centred ones tolerated by Western societies.

We will not analyse these premises extensively (even though they are not universally shared) as we would like to concentrate on a specific point: namely, that in many competitive and working contexts there are moral and social reasons to acknowledge the use of cognitive enhancers.

In this paper we will argue that these reasons are not strong enough to hinder the legal permissibility and moral acceptability of PCE. In fact, to prohibit their use would mean to go against individual freedom and reduce the benefits that they would bring to both the individual and the society. These effects cannot be justified by the problems caused by cognitive enhancement. However, introducing a duty of disclosure could be a good way to regulate PCE use, constituting a more confined limitation of freedom than prohibiting it completely. PCE use in specific contexts can be justified by the benefits that it would give, and the implementation of the disclosure duty could be realized with the same legal tools as a ban, without having its freedom limitations.

## Main text

### Cognitive enhancement and public interest

There are arguments in favour of the use of “smart drugs”, while others are completely against it [47]. Broadly speaking, the two positions have been defined in different ways depending on the context, but for the purpose of the present investigation – without going into further detail for lack of space – we will limit ourselves to calling *bio-liberal* those supporting the use of PCE and *bio-conservative* those against it [48]. Given the general mistrust in the enhancement project by the latter, for the purpose of the present work we will only focus on the former. This choice is due to two reasons: first, some restrictions to PCE use are more of a challenge for bio-liberals than for bio-conservatives, because bio-conservatives are by definition inclined towards a full-scale rejection of enhancers. Second, the bio-liberal paradigm seems to be more consistent with a pluralistic society such as ours, since it does not presuppose strong positions about human nature and leaves room for people to autonomously decide which principles to follow. If bio-liberals are persuaded to introduce some form of regulation, the latter will be all the more justified in the eyes of bio-conservatives, who are against enhancement. In this sense, there is no need for bio-conservatives to accept bio-liberal assumptions. In fact, the specific point here is that of the duty of disclosure. Bio-conservatives may find it too bland or insufficient, but they probably would not object to it as such, as they too think it might be better than a total deregulation. In line with this view, the liberal paradigm conventionally accepts that anyone can autonomously decide what substance to take and how, as long as this does not damage others – it could also be hypothesized that cognitive enhancement does not jeopardize the fairness of competition any more than many other techniques or socio-economical conditions that are generally seen as unproblematic [49, 50]. For example, some students may attend expensive public schools while others cannot. Some would-be lawyers or managers may meet influential people thanks to their familiar relationships while others cannot, and so on.

Within such paradigm, we think it is reasonable to argue in favour of a moderate liberal position implying a moral and social duty *to declare the use (or non-use) of cognitive enhancements*. We will suggest that it could prevent certain forms of harm to single individuals or even society as a whole. It is interesting to note that in the recent report of the Presidential Commission for the Study of Bioethical Issues (PCSBI) [51] -issued to give recommendations on the ethical use of medications and other types of “neural modification”- PCE have been integrated with other kinds of “neural interventions” such as deep brain stimulation, for instance. However, out of simplicity for both our argument and the availability and the implementation of the procedures, we will only focus on PCE in this occasion. Moreover, another of our premises is that, differently from previous accounts [7, 29, 52], we conceive of the use of enhancers as a responsible behaviour: it should not jeopardize the freedom or rights of others nor go against the public interest.

Our claim that PCE use should be disclosed is also due to equity reasons: especially in the future, PCE could allow for a much greater and quicker performance enhancement than any other presently existent technique, so that PCE users would be much more advantaged compared to non-users. In addition to this, while other “pre-existing inequalities” are often evident, public, or simply investigable, cognitive enhancement by means of a pill would likely remain “secret”. In this sense, there can be reasons related to authenticity [53] and transparency to be set against the respect for individual privacy. However, the fact remains that establishing an obligation to report the use of PCE seems to run against the liberal framework that we have just outlined. In particular, this duty seems opposed to the notions of autonomy and privacy involved in the liberal framework. Therefore, it is important to clarify in more detail the role of autonomy and privacy in our argument.

### Autonomy and privacy

Even though there are several definitions of it, the concept of autonomy “is generally understood to refer to the capacity to be one’s own person, to live one’s life according to reasons and motivations that are taken as one’s own and not the product of manipulative or distorting external forces” [54]. Those who are autonomous can decide for themselves without interference from others or personal limitations preventing significant choices; they can act according to a plan of their own, designed without constrictions. Autonomy also concerns the freedom to decide what to believe in and to weigh the pros and cons of a given course of action. Another fundamental element regards the awareness of the rules one establishes and follows. A central role in this is played by rational reflection, or the ability to assess existing traditions and norms of conduct, as well as the capability of choosing – with the necessary emotional detachment – which ones to follow and which to ignore. This specific version of autonomy is often defined as *competence* in medical-legal contexts. We could say, in other words, that being autonomous means being aware of the outcome of one’s deliberations and being driven by one’s own purposes. Being autonomous means being oneself, being driven by considerations, desires, conditions, and characteristics that are part of what can be considered the authentic self – which, as such, becomes an irrefutable value. 1

The concept of privacy generally regards the protection of a space of non-interference, based on a principle of “inviolate personality” which – as Warren and Brandeis famously stated [55] – is a part of a general right of immunity of the person. Indeed, privacy limits the intrusion into a person’s seclusion or solitude or into her private affairs. The right to privacy seems to prevent the public disclosure of embarrassing facts as well as publicly putting one in a bad light [56]. We argue that, if others can enter your own sphere, even just to see it, this potentially affects your own freedom of action, because being “observed” implies giving the others an advantage over us, thus creating an asymmetry between us and them, and generally binding and limiting us. The right to privacy thus seems an essential correlate of autonomy, which, as we have noted, is the preferred framework of liberal societies.

In the current cultural climate, adults above a certain threshold of competence are always presumed to be autonomous and to have the obligation to respect that autonomy. So, whenever one intervenes against an individual’s will or violates their privacy, one is also disrespecting that individual as a person. But then, is it possible to identify a principle that goes against individual privacy in the case of PCE? Is the use of PCE related to morality and the public interest? We will try to show that it is, also by means of a few examples.

### Unfairness and composition effects: some reasons to disclose the use of PCE

In order to support the duty of disclosure of PCE use, one must first of all clarify what properties they usually or at least frequently present, in accordance to what was found in the literature. Namely, “the enhancer has acute and/or chronic effects. In the first case, shortly after taking the drug the performance is significantly better than average; in the second case, there is a growing or lasting effect, which, however, is set to diminish when one stops taking the drug; those effects are significant (there is a visible difference between taking and not taking the drug) and sometimes dramatic; a third feature, not directly related to enhancers as such, is their varying safety, availability, and legal permissibility, which might either induce people to take them or refrain them from doing so”. 2 Recent reviews [57, 58] raise some doubts about the properties attributed to the PCE available today and by this fact conclude that the diffusion of enhancers should not constitute a particular social problem.

We think this is only partially true, since researchers are always looking for new and more effective techniques. A recent discovery regards a possible mechanism to make an adult brain as plastic as a child’s, which is more apt to form new synapses. The key is a protein present in brain cells known as PirB in the animal models. The main function of PirB is that of stabilizing neural connections. But this protection also makes them more rigid and less able to acquire new skills and information. By interfering with the normal function of PirB, it seems possible to quickly induce new neural connections in adult brains, which is a way to enhance cognitive processes, since one can learn more rapidly than usually, provided that scientists find a safe way to knock out the human variant of the molecule, called LilrB2 [59].

Moreover, a recent qualitative study showed that Adderall affect intellectual capacities like executive functions, working memory and information processing, but their use is also associated with significant changes in the user’s emotional states, which are as relevant as the cognitive ones. “Such alterations appear to be an important dimension of the drug effects that users perceive to enable improved academic performance” [26]. Participants said that a better emotional or affective state was the most important effect of the enhancers. According to this study, participants reported “feeling up”, a general increase in their energy levels (both physical and mental) and a strong sense of well-being; “drivenness”, a strong need to do something, as if they suddenly had extra energy; a strong desire to achieve a goal, or to complete a task; “interestedness”, enhanced abilities to become emotionally invested in issues related to their work, the perception of caring about those issues; and “enjoyment”, experiencing their work as a pleasurable activity. Feeling up was also described as “upness”: both energization and mood improvement. Drivenness was perceived as an internal push, pressure or motivation to get started with one’s work and to keep on it steadily, through interestedness.

One can therefore claim that the PCE’s emotional impact is one of the reasons for their use. As the authors of the quoted study openly admit, the fact that PCE can change one’s emotional state is “part of what makes stimulant drugs useful in relation to academic work” [26]. However, there is evidence that Ritalin and Adderall have a strong effect on the dopaminergic system: they influence attention, the system of pleasure and that of emotions, causing a euphoric effect [27, 28]. It should also be noted that, if cognitive enhancers are so popular in academia and school, it probably means that students and professors aim at feeling something relevant, which might not be only a cognitive effect [60].

Combining strictly cognitive effects with emotional-motivational ones, the overall effect of these off-label medications is very strong. Aside from the well-known “rat-race” argument [61] focused on showing the structural limits of the idea of being better thanks to PCE – as well as their intrinsic subordination to peer-pressure inputs [62] – there is another important issue that needs attention, which lies at the heart of our argument: the fairness towards the “unenhanced” and the potentially dysfunctional social consequences of undisclosed PCE use.

Indeed, two lines of argument can be proposed to support the need to disclose the use of PCE. The first has to do with the idea of fairness. Within the context of personal autonomy that we have chosen, it is defined as follows. In a competitive environment, where only one candidate gets the prize (say, a scholarship or a job), each participant is encouraged to try to improve their performance in the way they deem most effective, provided it complies with the rules in place, which are presumably known to all participants. The assumption of PCE, however, would be an extrinsic reinforcement whose effects are “hidden” and unlikely to be known by the other participants. A participant’s pharmaceutical cognitive enhancement would alter the initial situation of equality – which, however, could be easily amended by disclosing the use of PCE. In this case, the reference to the parity thesis [63] does not seem to apply, as PCE appear to be qualitatively different from common stimulants such as caffeine or nicotine. According to the parity thesis, which can be summed up with the expression “nothing new”, the simple fact that there is innovative technology, capable of direct intervention on the brain through neuroscientific knowledge, should not necessarily lead us to think that there will be significant or unheard-of problems. However, as we said earlier, there does not seem to be any analogy between stimulants such as caffeine and tobacco, and new molecules used to increase attention and concentration skills (for example, Modafinil and Adderall). The main difference is the targeted use people make of new molecules, and their action that, while still being under discussion, seems to be more effective [64]. In any case, the argument is also aimed to consider future developments in pharmacology, possibly leading to a more marked difference in quality and quantity of new generation stimulants compared to caffeine. The specificity of PCE also emerges from the fact that cognitive enhancement through specific types of PCE was proposed as compulsory for those engaged in professions which the entire community benefits from (e.g., surgeons, airline pilots) [46]. In that case, there would have been a duty to declare the use of PCE to control the quality standard of the professionals.

Finally, the effect of these substances varies depending on the subject, also in function of the dosage and other factors related to the context of use [30, 65]. In our opinion, there is a realistic risk that a reasonably high number of individuals -not knowing what drugs other people use- would be tempted to buy and use everything is available, given that competitors could use those drugs as well. There would thus be a random and disruptive effect over the performance of the participants, undermining the equality of opportunities.

The second line of argument is part of a consequentialist framework, as it refers to the potential composition effects of an undeclared use of PCE by *many* individuals. This argument supports the thesis that there is a social duty to declare cognitive enhancement. As we shall see, there are situations in which some researchers, workers or students who secretly use PCE can be considered more skilled and better at performing their task than they really are. But this is not the main problem per se. The potentially dysfunctional social consequences of PCE use are due to its being undisclosed. If an individual does not have her drug dose available, she will not be capable of the same performance as usual and, in some professions, might even put her colleagues at risk. If standards are set based on PCE-using workers, this may lead to an organizational dysfunction. In this case, the relative lack of respect for individual autonomy and privacy can be justified by utilitarian criteria. In fact, the benefit that the individual seeks in PCE could be made vain by the general negative consequences caused by the widespread use of PCE. The idea is that one should give up part of your autonomy/privacy for the sake of your group’s greater overall wellbeing.

We argue that in the relevant contexts – like open competitive exams, recruitment, high performance professions or public responsibility (from doctors to pilots and firemen) – preventive measures similar to those employed to forbid the use of specific substances should be implemented. In particular, we think the duty to disclose PCE use should be included among those rules that, if not followed, lead to disqualification. In addition to this, spot checks and other surveillance measures could be implemented to ensure that everyone who uses PCE communicates it to the relevant people in charge (examination boards, employers and supervisors). Such surveillance measures could include productivity indexes to evaluate anomalous variations, periodic or surprise blood tests, the ability to test the required skills even outside working hours. The mere possibility of implementing such surveillance measures, combined with sanctions, would likely lead to respecting the duty of disclosure. In this way the disclosure of PCE use would not become totally public and would have the advantage of limiting the restriction of individual privacy while gaining the maximum benefit for society as a whole.

The notion of PCE use duty of disclosure derives from the consideration that there can be conflicting rights. On the one hand there is the right of society to have vital services efficient and fair competitive conditions in some contexts that have consequences on the community. On the other hand, there are the rights of individuals to have their privacy and their autonomy protected. Compared to prohibition, the duty of disclosure seems to maintain the interests of society at the price of a smaller reduction of PCE consumer rights.

The idea of non-public disclosure means that not all have to know that an individual uses PCE, neither in the event that he can continue to take them in controlled conditions nor in cases he should suspend the assumption due to social needs (see examples below). In any organized situation, there are people who have the responsibility, according to coded procedures, to select and control staff. Only they can and must be aware of the disclosure required on schedule. The lack of communication, once discovered, could be a right cause for sanction or dismissal. In state organizations or professions in the public interest, which are already regulated by state laws (for instance, the medical profession), it is conceivable that there should be a legal enforcement, explicitly provided for, precisely on the grounds that are exposed in this paper. Periodic checks explicitly programmed in the form of anti-doping tests such as those used in sports, could be a fair method of implementation of the duty of disclosure. Obviously, these measures relate to substances that are not already explicitly prohibited, as the latter are regulated by the laws that already exist. However, periodic inspections could not be sufficient. One could hypothesize introducing spot checks, but these raise greater objections related to individual privacy and autonomy.

The duty of disclosure can be compared with other types of discourage-use policies on PCE. In particular, for non-prohibited substances, one can introduce monetary incentives not to use PCE without asking to reveal their use. Or one can increase the taxation on legal PCE to decrease their use without prohibiting them and without violating individual privacy. This measure seems to limit privacy and autonomy to a lesser extent, but is avoidable by purchasing PCE outside legal channels and, therefore, less effective. Monetary incentives are costly and do not guarantee compliance, especially in cases where the performance of the professional depends at least partly on the PCE. The fact that there are two distinct and convergent arguments related to PCE seems to indicate that the issue at stake is important. In this vein, we will now propose some compelling and realistic examples related to organizational and institutional contexts. These examples highlight the argument for the (moral and socially relevant) duty to disclose the use of PCE, insofar as it is sufficiently harmful or unfair to third parties (though not sufficiently harmful or unfair to justify a legal ban).

#### Example 1

During a public competition for the recruitment of senior civil servants, only a few candidates are left for the final selection. The last exam consists of an interview during which the candidates have to pass several tests. None of the candidates know the nature of these tests, but flair and self-confidence are said to be particularly appreciated. If there are no rules regarding PCE, each candidate will be tempted to take a cognitive enhancer that works in the short-term in addition to other mood-altering drugs, in particular tranquilizers and those that bring on euphoria. Since each candidate believes the exam to be decisive, they are likely to be tempted to use the most powerful drugs available (let us say, for sake of argument, Aniracetam), at the upper bounds of the suggested dose. Perhaps someone may be tempted to take an extra dose to beat the other candidates, putting his or her health at risk. Additionally, the self-assuredness and other performance enhancements brought about by a heavy dose of PCE during the recruitment process will not match the average level of performance that the candidate will be able to ensure after being hired.

This harms society, because it leads to selecting a civil servant whose expected performance does not match his or her actual performance, while other candidates could have been more efficient. If instead each candidate were required, prior to the interview, to declare what drugs they expect to take, then the recruitment commission would have all the necessary information for an exhaustive assessment (of course, some candidates could cheat with regards to the drugs, but to offset this, some sort of surprise drug test could be introduced). 3 Knowing what PCE are taken by some candidates would allow everyone to act in order to ensure they have the same opportunities while not putting their health at risk. Indeed, if someone were to decide to take excessive doses or use dangerous substances, the other candidates would be free not to do the same. This would prevent an “arms race” that would be morally inequitable, dangerous at the individual level, and inefficient at the social level.

#### Example 2

Steve and Laura work for the same company in the Silicon Valley. They have a similar background and play a similar role at work. When the head of their department switches to another company, this opens the possibility for one of them to fill the vacancy. The company chooses to carry out an internal competition to decide who between Steve and Laura is the best candidate for the post of head of department. Steve and Laura are granted a two-week vacation to study and prepare for the best. They do not know how the test will take place and assume it is a traditional examination. The company has decided instead to evaluate both the content of the answers and the speed at which the candidates give those answers. Before the exam, Steve takes a 15 mg Adderall tab in order to improve his cognitive performance during the test. To be fair, he offers the same tab to Laura, but she refuses to take an enhancer. At the end of the examination, the post is given to Steve. In fact, Steve answered fewer questions, but was much faster than Laura. The company’s decision is due to the fact that it wants both competence and quick thinking. Laura is very upset: she thinks that the decision is very unfair. If she had taken Adderall, her speed in responding would have been comparable to Steve’s and therefore would have got the job. Laura therefore asks Steve to confess that he was under a PCE. Steve, however, refuses. He first argues that he had given Laura the opportunity to use a PCE herself, but she chose not to take the enhancer. Second, Steve points out that there is no requirement to disclose the use of PCE. Otherwise, why has Laura not revealed that she had been drinking a lot of coffee before the exam? 4 Here, it would appear as if the duty to disclose the use of PCE would be useful to reintroduce a neutral judge in the assessment of the ability of the two otherwise morally sound candidates. The establishment of a clear way of evaluating the enhancers will be accepted by both individuals –as they both believe to be right. Speculations related to a number of aspects of the example are relevant of course (i.e. in the US, Adam’s offer to Eve could be a punishable offense, with a legal sanction of up to 2 years in prison depending on the specific state), 5 but we urge the readers to leave momentarily aside these practical realities of how the law is currently dealing with PCE, to focus instead on the interpersonal impact that the undisclosed use of them could and does have in competitive contexts and imagine that, for the sake of the argument, this example takes place in a country where the use of Adderall is legal. Yet, our examples aim at being as universal as possible, so we believe that including some specific variables of one country over another would be counterproductive for a global analysis of the issues at stake. The main issue raised in the example is the fact that PCE use affects certain aspects of recruitment tests, such as the speed in responding, but not others, such as knowledge of a specific subject. One could get around this problem by changing the tests so as to give less importance to the aspects influenced by PCE, but that would mean changing a process that in normal conditions has proved effective, and it would be costly. The duty of disclosure of PCE use, instead, solves the problem in a much more cost-effective way.

#### Example 3

Sumeet has always wanted to help his neighbour. As a boy he wanted to be a doctor, but his family could not afford to pay the university fee. So, after high school, Sumeet decided to work in the public ambulance service. Sumeet has never been very good at school, but he thinks he could be a great ambulance driver, thus helping to save many lives. The selection for the job involves a written test, which worries him a lot. Talking with a friend he learns about Ritalin, which is not prohibited and could help him pass the test. Sumeet, however, knows that the purchase of Ritalin requires prescription. The friend then comes to his aid, and gives him the tabs. Sumeet, hoping they will help him focus and be sharper, takes them before the written test. The examination goes well and Sumeet chooses to use the remaining tablets in the oral test, at which he performs very well. He is then hired as an ambulance driver.

Sumeet realizes that if he continues to take Ritalin when in service, his driving performance will be better. One evening, just when Sumeet has run out of Ritalin, there is a terrorist attack in his city. Without Ritalin, Sumeet feels weak both on the psychological and on the physical level: his reflexes are slower, and in one case he even takes the wrong way. The doctor with him sees that there is something wrong and tries to reassure him. Sumeet is thus able to complete his shift without causing major damage. But his supervisor scolds him: certainly he could have done much more, given the standards he had shown previously. So Sumeet, who thinks he has nothing to hide, reveals the cause of his poor performance. On hearing this, his supervisor gets extremely angry. Not only did Sumeet “cheat” the exam, but above all he put at great risk the injured and the ambulance doctors. The supervisor should know whom to count on in difficult times. And if a driver varies its performance according to the PCE he takes or does not take without the knowledge of his colleagues, then the whole team work can be severely compromised. Sumeet is then fired, but he does not understand why: he thinks that he only took Ritalin to do his job better. 6

One could argue that the problem here is mainly linked to Sumeet’s lack of information on the effects of Ritalin, and that therefore the solution to the problem comes especially from providing publicly accessible information [66]. In fact, as in any human activity, it is unrealistic to think that all people are always perfectly informed. Therefore, the duty of disclosure is to offset this potential lack of information. The duty of disclosure of PCE use would obviously be a way to convey information, because, as mentioned earlier, the breach would have significant consequences for the worker. Therefore, the worker would be likely motivated to make detailed inquiries regarding the rules on the use of PCE, which presumably she would not do otherwise. Also, those who knew that Sumeet uses Ritalin could inform him of the risks and consequences of it. So this would create a situation where the amount of information available is greater, because the controllers would be informed about Sumeet’s condition and Sumeet would be informed about what Ritalin can do.

#### Example 4

An important goal for a school system is to provide equal opportunities to students in any region of their country. To do this one needs to evaluate schools in different areas to find out if and where to intervene to improve quality so as to bring all areas to the level of other ones. To this purpose, the Organisation for Economic Cooperation and Development performs sample tests on students in many countries. One of the means to measure the students’ skills and knowledge is the Programme for Student Assessment (PSA). Imagine the situation that might arise in the classroom where students are old enough to legally take cognitive enhancers. Even if the tests are anonymous for individual students, the result of the classes or schools is known. Teachers could then push their students to take PCE so as to perform well and the teachers themselves look good. As mentioned, cognitive enhancers do not improve the intelligence nor allow one to answer a question one does not know the answer to.

However, the PCE may improve test performance and make it so that in certain schools or in certain regions of the country there are high scores that do not correspond to the quality of the teaching methods or the actual ability of the students. Consequently, the data would not be correctly interpreted and no efficient decision could be taken on their basis (it should be remembered that these are sample tests and therefore few classes can be used to judge the level of preparation of an entire region). The results thus distorted by the use of PCE could lead authorities to extend the teaching methods of the well-performing schools, as they would be considered the most effective when in fact they are no better than others. Thus many resources would be wasted and really effective teaching methods would be neglected. A further consequence would be the lack of action in favour of schools or areas where economic or social deficiencies should be remedied to improve the quality of education, but where, because of the use of PCE, it seems that school results are good and that therefore there is no need for any state intervention. In this case, there would appear to be both a moral duty and a social interest in reporting the use of PCE. Indeed, the use of PCE could be deemed fully legitimate and, if openly reported, it could become an additional element in the assessment of instruments suitable for improving school performance (that is, the main purpose of the tests in question). By knowing what classes, in what geographic areas, took cognitive enhancers, it will be possible to assess their effectiveness without introducing biases in the overall results.

This is of social interest as it helps with the efficient allocation of resources. In fact, there also seems to be a moral duty, since teachers who encouraged their students to take PCE without reporting it might be trying to hide their shortcomings as educators. Instead, taking PCE unbeknownst to evaluators may result in the failure to identify situations of economic or cultural distress, because school performance has improved only thanks to the use of cognitive enhancers. In other words, teachers or students from the schools chosen for sample testing in a given region may be trying to portray themselves in a positive light, but by doing so they would cause harm to all the other schools in the region, where school performance is poor and investments on the part of the state are surely needed.

It could be argued that the students’ use of PCE is positive for society, as it increases the stock of efficiency of the entire community [67]. This might be true if PCE use were made compulsory, which seems to have strong contraindications in itself [68]. If the use was only allowed without the duty of disclosure, so that some classes would have many users and others just a few, there would be the problems that we have highlighted in this example. In other words, the fact that teachers can skew the results of a few school classes chosen as a sample, thanks to the use of PCE recommended to their students, may lead to erroneous assessments of entire school districts, with the consequent allocation of resources on the basis of results which do not correspond to the actual level of overall preparation of the students.

#### Example 5

In some cases, a candidate for a job may resort to the use of cognitive enhancers to increase his performance in tests and interviews. In particular, the candidate in question may resort to drugs that would raise his mood (such as fluoxetine) and/or real enhancers that would improve single aspects of his cognitive functioning. In these situations, as it often happens, tests and interviews may not be targeted to the specific task that the candidate will have to perform, but only to assess his/her general skills, both because of the difficulty of making a targeted selection and because of the choice to hire “generally good” staff to train later according to specific needs. There are two problems that can arise in such cases. Firstly, attention-increasing neuro-stimulators seem to have the side effect of decreasing creativity and/or other mental functions [63]. Evidence shows an inverse relationship between concentration and creativity, understood as the ability to find new, alternative ways to tackle a task that requires a solution. The hiring organization would therefore be selecting an employee who might have a performance-related imbalance induced by enhancers (besides, after the selection he could be induced to keep taking them so as to maintain the standards with which he passed the selection process). The company could not resort to the candidate’s real strengths, risking entrusting him with an unsuitable task.

Secondly, the use of a cosmetic pharmacology of a euphoric kind, especially at high doses, tends to produce a professional conduct characterized by optimism, overestimation of one’s own abilities and underestimation of the risks – regardless of data [69–71]. This can produce a professional figure that may be likeable in the early stages but, in the long run, is likely to be counterproductive and even dangerous. One example is that of financial operators, but also surgeons and all workers who are in the control of safety equipment. While, admittedly, a depressive personality is just as dysfunctional as regards these tasks, the supervisors’ knowledge of the worker’s mood-improving drug use would give more elements for a better distribution of tasks and responsibilities in the workplace, with a positive impact not only for the specific organization but also for society as a whole.

## Discussion

To sum up, the five examples we made are aimed to illustrate various situations in which the duty of disclosure can help solve problematic issues. In Example 1, public competitions for recruitment of civil servants might be affected by the fact that only some candidates have taken PCE. In this way, it may happen that the recruiter would select the latter people and not those who are actually best fit for the job. The duty of disclosure might solve this problem without resorting to a ban of PCE. In Example 2, we have shown how interpersonal competition can be affected by PCE, in that they influence certain aspects (such as quick responses) but not others (such as knowledge). The duty of disclosure can avoid this risk. In Example 3, the duty of disclosure induces people to know more about the effects of PCE use, especially for those whose profession affects others in a direct and significant way. Also, it helps to avoid dangerous situations due to poor performance on the part of PCE users when they are not on PCE – in such situations, in fact, their superiors or their colleagues would not be able to intervene as they would not be aware of the workers’ habitual PCE use. In Example 4, the situation shown in Example 1 has much more serious social effects, because if students chosen as a sample to assess the school system make use of PCE, this can lead to allocate public resources in a way that does not reflect the truth. Once again, the duty of disclosure might solve this problem. Finally, Example 5 recalls Example 1 but magnifies the potential problems related to recruitment in cases of undisclosed PCE use. In fact, someone hired while on PCE could be expected to always perform in a way that actually depends on their PCE use. Also, PCE accentuate certain traits that may cause workers to overestimate their own capabilities, with disastrous effects on their performance, which in many cases will have consequences on the public. The duty of disclosure avoids these distortions.

The purpose of these examples is to underline the tension between the individual’s use of PCE and some practical problems related to a concealed use of such enhancers in a liberal society. 7 Unintentional consequences, in fact, may produce sub-optimal situations for society as a whole.

Our point is to underline overlooked difficulties within the debate on smart drugs. Although on the one hand the unrestricted use of PCE preserves the principle of personal autonomy that (at least at the theoretical level) should be given priority in liberal societies, on the other hand, the consequences – be they intentional or not – can lead to unfair outcomes or social situations that clash against some other principles dear to liberalism. In particular, as noted above, there may be limitations to the principle of equal opportunities. Also, the use of PCE in complex social contexts is likely to produce unequal or dysfunctional results for the group. Thus – based on the realistic examples that we provided – one might justify the practice of *posing some limitations to personal autonomy related to the use of PCE* through a restriction of the subject’s privacy in competitive contexts. Indeed, if one has to reveal to controllers that one makes use of PCE, this disclosure may have consequences: increased surveillance by one’s superiors, a request to use or not to use PCE in certain situations, or even a different view of one’s work. This is obviously a limitation of one’s autonomy as a person who wants to manage their work performance without interference, albeit within a general regulatory framework.

The legitimacy of introducing a duty of disclosure can be supported by values that are deeply entrenched in liberal societies. It suggests a restriction of individual freedom/autonomy if the exercise of the latter puts in danger the freedom/autonomy of others, as it happens in the case of the concealed use of PCE.

Reasons related to equity and social interests, therefore, suggest that there is a moral and social duty to acknowledge the use of cognitive enhancing drugs in competitive-selective contexts. As we have already noted, in a context in which personal autonomy has priority, these reasons are not strong enough to claim that PCE use as such is morally unacceptable. Furthermore, considering that some PCE are prohibited by the law, as explained above, the reasons that lead us to propose the duty of disclosure do not directly affect the question of legislation, which often refers to health problems or public order issues that are not here considered.

In general, prohibiting the use of PCE per se would amount to a privation of freedom/autonomy and a reduction of the benefits that they could bring to the individual and society, which is not justified so far by the problems caused by cognitive enhancement. However, introducing a duty of disclosure could be both morally acceptable and legally feasible, and it would constitute a more confined limitation of freedom than prohibiting PCE use in the context in which autonomy is the first good to consider. PCE disclosure in specific contexts can be justified by the reduction of the negative effects of an uncontrolled use of PCE and by the benefits that a controlled use of PCE would give. In the absence of serious health hazards produced by the substances in question, the autonomy of individuals can be considered the number one concern in the face of some moral objections to the use of PCE. But other considerations that here we have tried to develop raise a number of potential problems at the aggregate level created by uncontrolled use of PCE. The examples we have described draw some scenarios in which these problems occur with greater impact. Consequently, it can be argued that the duty of disclosure, in the form proposed by us, is more respectful of the autonomy of individuals than the prohibition of PCE use as such.

## Conclusion

In this paper we have tried to show that there is tension between the personal use of PCE and the unintended social consequences of that use. To shed light on this important issue related to an increasingly widespread form of cognitive enhancement, we have proposed five examples. What has emerged is that we value autonomy and privacy and think that they should be respected in the most effective way. However, in a liberal society, a partial reduction of our right to privacy might be necessary in order to preserve the equally important principle of fairness as well as social safety and efficiency - which an unregulated use of PCE could threaten. The legitimacy of this prevision derives from other values deeply entrenched in liberal societies, such as the restriction of individual autonomy if it endangers that of others. Reasons related to equity and social interests, therefore, suggest that there can be a moral duty to publicly acknowledge the use of PCE in order to limit such potential damages. Indeed, there are situations in which the use of stimulants might improve the user’s condition while having composition effects that turn out to be harmful for the group of reference or even society as a whole. Many people think that citizens should be free to use PCE, and banning them is not a viable option, as it goes against the principle of autonomy. However, as we have suggested in this paper, a via media could be found in the duty of disclosure.

### Ethics approval and consent to participate

Not Applicable.

### Data availability

Not Applicable.

## Abbreviations

CE: Cognitive Enhancers
PCE: Pharmaceutical Cognitive Enhancers

## Footnotes

1 One of the authors has analysed the concept of autonomy and some of its controversial interpretations in a number of contexts that could provide further insights to those interested in the topic, but we decided not to include such considerations in this work for a matter of focus [72].

2 Such a scheme has been discussed elsewhere, but here the issue is looked at from a slightly different angle [73].

3 It should be acknowledged that the advantages of the duty to disclose policy we outline are contingent on the implementation of effective control measures The feasibility of these surprise tests, their effectiveness and their implementation will not be addressed here, but suffice to say that we are not the only ones concerned with ways of bringing to the surface our “biochemical status” in relation to a test or job interview. For instance, Barbara Sahakian [7, 9] has been a fervent supporter of the introduction of tests aimed at detecting the use of PCE by university students [74–76].

4 It should be noted that this argument is often used by bio-liberals: given that caffeine is also a cognitive enhancing substance, why should we limit the use of one enhancer over another? For a more precise analysis see: Julien, Robert M., Claire D. Advokat, and Joseph E. Comaty [77]. In particular 361-395.

5 We are particularly indebted to one of the reviewers for underlining the importance of this acknowledgment.

6 A similar motivation could be applied to the renowned cases of cocaine use among surgeons around the world. However, our intention is to focus on PCE precisely because – unlike cocaine – they are not *illegal* drugs.

7 In fact, we have not considered contexts (e.g. the army) in which such practices might be strongly encouraged or even required by the authorities [78]. Provided that someone’s choice to enter such a context (say, the army) is expected to have been made competently and without coercion, even though the compulsory use of PCE might be seen as limiting the individual’s autonomy, the fact that it is compulsory for *everyone* guarantees a fairer treatment to *all* the participants. Civil situations, instead, call for a different kind of attention on how to preserve fairness.
